# Isolation, identification and expression analysis of salt-induced genes in *Suaeda maritima*, a natural halophyte, using PCR-based suppression subtractive hybridization

**DOI:** 10.1186/1471-2229-9-69

**Published:** 2009-06-05

**Authors:** Binod B Sahu, Birendra P Shaw

**Affiliations:** 1 Environmental Biotechnology Laboratory, Institute of Life Sciences, Nalco Square, Bhubaneswar, PIN-751023, India

## Abstract

**Background:**

Despite wealth of information generated on salt tolerance mechanism, its basics still remain elusive. Thus, there is a need of continued effort to understand the salt tolerance mechanism using suitable biotechnological techniques and test plants (species) to enable development of salt tolerant cultivars of interest. Therefore, the present study was undertaken to generate information on salt stress responsive genes in a natural halophyte, *Suaeda maritima*, using PCR-based suppression subtractive hybridization (PCR-SSH) technique.

**Results:**

Forward and reverse SSH cDNA libraries were constructed after exposing the young plants to 425 mM NaCl for 24 h. From the forward SSH cDNA library, 429 high quality ESTs were obtained. BLASTX search and TIGR assembler programme revealed overexpression of 167 unigenes comprising 89 singletons and 78 contigs with ESTs redundancy of 81.8%. Among the unigenes, 32.5% were found to be of special interest, indicating novel function of these genes with regard to salt tolerance. Literature search for the known unigenes revealed that only 17 of them were salt-inducible. A comparative analysis of the existing SSH cDNA libraries for NaCl stress in plants showed that only a few overexpressing unigenes were common in them. Moreover, the present study also showed increased expression of phosphoethanolamine N-methyltransferase gene, indicating the possible accumulation of a much studied osmoticum, glycinebetaine, in halophyte under salt stress. Functional categorization of the proteins as per the Munich database in general revealed that salt tolerance could be largely determined by the proteins involved in transcription, signal transduction, protein activity regulation and cell differentiation and organogenesis.

**Conclusion:**

The study provided a clear indication of possible vital role of glycinebetaine in the salt tolerance process in *S. maritima*. However, the salt-induced expression of a large number of genes involved in a wide range of cellular functions was indicative of highly complex nature of the process as such. Most of the salt inducible genes, nonetheless, appeared to be species-specific. In light of the observations made, it is reasonable to emphasize that a comparative analysis of ESTs from SSH cDNA libraries generated systematically for a few halophytes with varying salt exposure time may clearly identify the key salt tolerance determinant genes to a minimum number, highly desirable for any genetic manipulation adventure.

## Background

Abiotic stresses are the principal cause of decreasing the average yield of major crops by more than 50% leading to losses worth hundreds of million dollars each year [1]. Among these, high soil salinity, contributed largely by Na^+ ^and often compounded with drought, is the main factor that adversely limits the growth and productivity of the major crop plants, including rice. Nevertheless, plants do exist in nature, like the halophytes, which survive and grow under extreme of salinity; severe climate changes throughout millions of years have resulted in the evolution of flora that exhibit substantial genetic diversity for adaptation to environmental perturbations [2]. It is in fact also believed that the genetic diversity in glycophyte, particularly in the crop plants, has been narrowed down over the millennia because of loss of alleles contributing significantly to salt adaptability [2]. Hence, while there is a need to understand the plants' response to salt stress, and the salt tolerance mechanism itself, with the common aim of enhancing salt tolerance in the crop plants, it is necessary that such attempt should include preferably the halophytic species. This is required, as variation in salt tolerance in the crop plants is relatively small, although working with the crop species has direct implication for agriculture.

Decades of research on the effect of salinity on growth and development of various plants and their response to salinity treatment at the physiological and biochemical levels has generated a wealth of information on the salt tolerance related parameters or salt tolerance determinants in plants. These may be grouped into 1) morphology adaptation, reflected as thickening of the leaves and cuticular wax deposition [3], 2) osmotic adjustment, reflected as accumulation of compatible solutes in the cytoplasm [4], 3) maintenance of ion homeostasis, reflected as H^+^-pump functioning [5], K^+^/Na^+ ^selectivity [6] and Na^+ ^exclusion and compartmentation [7-9], 4) cell signalling and gene expression, reflected as abscisic acid (ABA) and jasmonic acid (JA) accumulation [10,11], regulation of salt overly sensitive gene-1, *SOS1 *[12,13], Ca^2+^-induced increase in K^+^/Na^+ ^selectivity [14], increase in CDPK (Ca^2+^-dependent protein kinase) and MAPK (mitogen-activated protein kinase) activities [15,16] and synthesis of many transcription factors [15,17-19], 5) oxidative stress mitigation, reflected as activation of the antioxidative machinery [20,21], and 6) molecular trafficking and cell stability, reflected as the accumulation of heat-shock proteins (HSPs), jasmonic acid-induced proteins (JAIPs) and late embryogenesis abundant (LEA) proteins [15,17,22-25]. Although transgenic plants have been developed for many genes upregulated under salt stress, such as *P5CS (*Δ^1^-pyrroline-5-carboxylate synthetase), DNA helicase, carbonic anhydrase (*CA*), glyceraldehydes-3-phosphate dehydrogenase, Na^+^/H^+ ^antiporter [26-30], and the plants show enhanced tolerance to salinity, the field trials of many of them have remained highly unsuccessful [31]. Hence, the basics of salt tolerance still remain illusive, and needs further investigation.

The plant stress adaptive responses include dynamic transcriptome changes, presumably playing important role in co-ordination of the many different molecular events responsible for cellular and organismal homeostasis. These changes are generally regulated by complex signalling pathways, which are activated in response to various abiotic and biotic stimuli allowing the plants to cope with the changing environmental conditions [32]. There also occurs crosstalk between different signalling pathways [33,34], and identification of the convergent and divergent pathways between salinity and other abiotic stress responses and the nodes of signalling convergence may greatly enhance the understanding of the salinity stress response and the salt tolerance mechanism. Although several studies have been carried out on abiotic stress responsive signal pathways [15,35-37], and several reports exist on massive changes in the profile of gene expression in plants [38,39], these are mostly on *Arabidopsis *or other glycophytes, which are sensitive to salt. Such studies on the native flora of saline environment, i.e. halophytes, are scarce, although better information on the salt tolerance determinants is likely to come from the work on these plants rather than the work on the glycophytes.

One of the techniques being largely used to identify stress-responsive genes is subtractive hybridization. Attempts have been made to identify the salt stress regulated genes by suppression subtractive hybridization (SSH) in rice [19] and tomato [18]. However, to the best of our knowledge, the technique has so far not been used to identify the genes differentially expressing under salt stress in salt-tolerant plants. Among the salt-tolerant photosynthetic organisms, nevertheless, the salt-stress upregulated ESTs have been cloned in an alga, *Dunaliella salina *[40]. However, only a few highly upregulated ESTs were sequenced for further studies. Hence, the present work was carried out with the aim of generating cDNA library of salt-induced genes in *S. maritima*, a natural halophyte, following PCR based SSH in order to get information on salt stress response in the plant at the transcript level. Moreover, most of the salt-stress upregulated ESTs were identified so as to get a comprehensive picture of the salt-stress response in the plant at the level of gene, which might be useful in elucidating the molecular mechanism underlying salt tolerance.

## Methods

### Test plant and stress application

Seeds of *Suaeda maritima *L. were collected from the adult plants growing along the mangrove coastal belt in Orissa, India. The surface-sterilized seeds were soaked in de-ionized (Milli-Q) water overnight, transferred over wet filter paper in a petriplate and kept at 25°C for germination. It took approximately six days for the cotyledonary leaves to emerge fully. The germinated seeds were transferred over net, which remained in touch with half-strength Hoagland's solution contained in 150 ml plastic beakers. The Hoagland's solution contained 5.0 mM KNO_3_, 7.0 mM Ca(NO_3_)_2_, 2 mM MgSO_4_, 2 mM KH_2_PO_4_, 26 μM Fe-EDTA (Ethylenediaminetetraacetic acid Fe-salt), 45 μM H_3_BO_3_, 0.4 μM CuSO_4_, 0.7 μM ZnSO_4_, 9.1 μM MnCl_2_, 28 mM FeSO_4 _and 0.1 μM (NH_4_)_6_Mo_7_O_24 _(pH 5.7, adjusted with 1 M KOH). The seedlings were allowed to grow hydroponically in a growth chamber maintained at 24 ± 3°C, 70–75% relative humidity and 14 h light (200 μmol m^-2 ^s^-1^)/10 h dark cycle. The level of the medium was maintained by adding Milli-Q water. After 20 days, the seedlings were approximately 2 cm in height. At this stage, the seedlings were transferred to soil in plastic pots of known volume. The seedlings were set to acclimatize and grow for ~3 weeks under natural day/night cycle in a green house maintained at 24 ± 3°C and 70–75% relative humidity. During this period, the seedling attained a height of ~6 cm with lateral branches. The individual pots were watered every day alternately with approximately 150 ml of 1/10^th ^Hoagland's solution or Milli-Q water except on the penultimate day of the stress application. For the stress application, initially 100 ml of 0.5% NaCl, prepared in 1/10^th ^strength Hoagland's solution, was poured into the individual pots in the evening. The control pots received only Milli-Q water. After incubation for 1 h, another 150 ml of 1/10^th ^strength Hoagland's solution containing 5.75 g NaCl was poured into the treatment pots, raising their final NaCl treatment concentration (in 250 ml treatment volume) to 425 mM. It was determined earlier that 100 ml water was completely absorbed by the soil in the pot, while the additional 150 ml was partly absorbed and the rest inundated the soil. After 24 h of the initial NaCl treatment, the leaves of the seedlings were harvested, and were preserved in liquid N_2 _until further analysis. The leaves from the control plants were also preserved similarly. The treatment duration was determined based on the observation that the activity of the plasma membrane (PM) H^+^ATPase, involved in the maintenance of ion homeostasis, increased to a maximum in 30–36 h of the initial NaCl treatment. Although change in transcription, both quantitative and qualitative, in a plant can be noticed in less than half an hour of change in the environmental condition, a long duration exposure (24 h) of the plant to NaCl was preferred thinking that it would provide information about those genes that are really needed for adaptation of plants to saline environment in long run. Moreover, as the time gap between transcription and translation is generally 3 h or more, it was decided to go for RNA extraction after exposure (to NaCl) of the plant for 24 h, 6 h ahead of the exposure time at which the enzyme (PM-H^+^ATPase) activity reached to the maximum.

### RNA isolation and cDNA preparation

Total RNA was isolated from the leaves of control and 425 mM NaCl exposed plants following LiCl method [41]. mRNA was purified from the total RNA isolated using PolyATtract^® ^mRNA Isolation System I (Promega, USA) following the protocol supplied along with the kit. Double stranded cDNA was prepared by reverse transcription of 4 μg of the purified mRNA in 20 μl reaction solution following the steps outlined in the cDNA preparation kit (Super SMART PCR cDNA synthesis kit, Clontech, Palo alto, USA). The total RNA isolated from the leaves of both the control and NaCl-treated plants were processed simultaneously for the mRNA purification and cDNA preparation.

### Construction of SSH cDNA libraries

The SSH (Suppression Subtractive Hybridization) cDNA libraries, forward and reverse, were prepared using PCR-select-cDNA SSH kit (Clontech, Palo alto, USA). For this, the double stranded cDNAs prepared from the control and NaCl treated samples were digested separately with RsaI for 1.5 h to produce blunt ends. The digested products were extracted with phenol:chloroform:isoamyl alcohol (25:24:1), followed by extraction of the resulting aqueous phase with chloroform:isoamyl alcohol (24:1) twice. Finally, the digested cDNAs in the upper aqueous phase were ethanol precipitated and resuspended in nuclease free water (Promega, USA). The RsaI digested cDNAs of the control (C) and NaCl-treated (T) samples were divided into 4 equal parts. One part each of the C and T cDNA populations were ligated separately with adapter-1 (supplied in the SSH kit) at the 5' end in the reactions carried out overnight at 16°C, and the ligated products were called CA1 (RsaI digested cDNA population of control sample with adapter-1) and TA1 (RsaI digested cDNA population of NaCl-treated sample with adapter-1), respectively. Another part each of the C and T cDNA populations were ligated with adaptor-2R (supplied in the SSH kit) at the 5' end in a similar fashion, and were called respectively C2R (RsaI digested cDNA population of control sample with adapter-2R) and T2R (RsaI digested cDNA population of NaCl-treated sample with adapter-2R). The ligation of both the adaptors was checked by PCR amplification of the actin gene using actin gene-specific reverse primer (5'TTGCATCACTCAGCACCTTC) and adapter-specific forward primer (provided in the SSH kit). The remaining two parts of both C and T, representing the RsaI digested cDNA population with blunt end of the control and NaCl-treated samples, respectively were kept as such.

To create the forward SSH cDNA library, which would represent enriched population of the overexpressed and newly induced transcript messages, TA1 and T2R were considered as 'Tester-A' and “Tester-B', respectively, and the C as the 'Driver'. The opposite was the case for the creation of the reverse SSH cDNA library representing enriched population of the down-regulated transcripts, i.e. CA1 and C2R were considered as 'Tester-A' and 'Tester-B', respectively, and the T as the 'Driver'. Two rounds of hybridization were performed. In the first round, the denatured 'Tester-A' and 'Tester-B' were mixed separately with excess of the denatured 'Driver'. This resulted in subtraction of the cDNAs representing the less or equally abundant transcripts in the 'Tester' source (the sample considered as 'Tester') compared to that in the 'Driver' source (the sample considered as 'Driver'). Besides, this also resulted in the formation of single stranded cDNAs having adapter-1 (in the case when the 'Tester A' cDNA population was hybridized with the 'Driver') or adapter-2R (in the case when the 'Tester B' cDNA population was hybridized with the 'Driver'). These represented the transcripts not present in the 'Driver' source or present in greater number in the 'Tester' source than that in the 'Driver' source. In the second round of hybridization, the 'Tester-A' and 'Tester-B', hybridized previously with the excess of 'Driver' separately, were mixed together without denaturing, followed by mixing with excess of the denatured 'Driver'. This resulted in the formation of hybrid double stranded cDNA (one strand having adaptor-1 and the other strand having adaptor 2R at the 5' end) for those transcripts present only in the 'Tester' source or present in greater number in the 'Tester' source than that in the 'Driver' source. Two rounds of PCR were carried out with two different sets of primers specific to the two adaptors (supplied in the SSH kit) to exponentially amplify the hybrid cDNAs. The primary PCR was performed with one set of primers for 27 cycles (94°C for 3 minute followed by 27 cycles of 94°C for 30 seconds, 50°C for 30 seconds and 72°C for 45 seconds, and finally incubation at 72°C for 10 minutes and storage at 4°C forever). This was referred as the forward or the reverse subtracted SSH cDNA (library), as the case may be. The secondary PCR was performed with the other set of primers for 20 cycles maintaining the same conditions using ten-fold diluted product of the primary PCR. The secondary PCR products of the forward and the reverse SSH cDNA libraries were purified using Qiagen column, cloned into pGEMT EasyVector (Promega, USA) and transformed into JM109 *E. coli *competent cells.

The transformed bacteria for both the forward and the reverse SSH cDNA libraries were plated separately on four LB agar plates (15 μl SSH cDNA each plate), incubated at 37°C for 24 h, and the white colonies were picked-up. Approximately 500 colonies from both the forward and the reverse SSH libraries could be picked-up. These colonies were grown individually in liquid LB medium at 37°C overnight at 200 rpm in 96 well plates. The medium contained 10% glycerol to facilitate long period storage. Inoculums of the individual culture were then grown in 2 ml of the same medium (supplemented with 100 μg ml^-1 ^ampicillin) at 37°C and 200 rpm overnight. The plasmids were isolated using Qiaprep Spin Mini-Prep kit (Qiagen, GmbH) as per the manufacturer's protocol.

### Screening and authentication of the SSH libraries

Randomly selected 150 plasmid samples of each library were spotted (approximately 50 ng plasmid DNA each spot) separately on 8" × 10" nylon membrane (N^+^, Amersham Hybond) in duplicate. The secondary PCR products (100 ng, prepared afresh) of the forward and the reverse SSH cDNA libraries were labelled separately with α-^32^P-dATP by random primer labelling as per the instruction of the SSH screening kit (PCR-select screening kit, Clontech), and purified by BioRad spin-30 column (Bio-Rad, USA). The plasmid spotted membranes were incubated separately for half an hour in 30 ml prehybridization buffer (7% SDS and 10 mM Na-EDTA in 0.5 M sodium phosphate buffer, pH 7.2) at 65°C in 300 mm × 35 mm hybridization bottles. The buffer in each bottle was replaced with 30 ml of fresh prehybridization buffer, maintained at 65°C. The desired denatured probe was added to the individual bottles and hybridization was allowed to continue overnight at 65°C. One of the two membranes spotted with the plasmids from the forward SSH cDNA library was hybridized with the probe prepared from the secondary PCR product of the forward SSH cDNA library and the other with the probe prepared from the secondary PCR product of the reverse SSH cDNA library. Similarly, one of the two membranes spotted with the plasmids from the reverse SSH cDNA library was hybridized with the probe prepared from the forward SSH cDNA library and the other with probe prepared from the reverse SSH cDNA library. After the hybridization reaction, the membranes were washed with 30 ml of wash buffer-I (1 × SSC, pH 7.0 containing 150 mM NaCl, 15 mM Na_3_Citrate.2H_2_O and 0.1% SDS) for 30 min at 65°C followed by washing with 30 ml of wash buffer-II (0.5 × SSC and 0.1% SDS) at 65°C for 15 min. The membranes were air-dried and exposed to X-ray film at -70°C overnight, and developed.

### Sequencing and analysis of the cloned ESTs

The plasmid inserts of only the forward SSH cDNA library were considered for sequencing. The plasmids purified by Qiagen mini-prep plasmid kit were sent for single pass sequencing at The Centre for Genomic Application (TCGA, New Delhi) with SP_6 _as the forward primer. The sequences obtained were fed into VecScreen software (NCBI) to remove the vector sequence contaminations. The sequences of the adaptors were removed manually. The expressed sequence tags (ESTs) of approximately 100 bp or more in length were only considered for further analysis. The EST sequences were grouped into singletons and contigs using TIGR assembler  and were termed as unigenes. The unigene sequences were blasted for homology search using BLASTX programme (default) at NCBI database, and categorised into the proteins with known function, the proteins with unknown function and the proteins with no match in the database. The unigenes were then grouped into functional categories using MIPS (Munich Information for Protein Sequences) function catalogue  developed based on the information available on the function of a protein in *Arabidopsis thaliana *protein database. For this, the unigenes were individually assigned a unique locus name after the BLAST against the *A. thaliana *protein database. The locus names were fed to the MIPS functional catalogue and the genes were clustered under different functional categories.

### Expression validation by Northern and real time PCR (qRT-PCR)

Slot blot Northern analysis was done for select EST clones of the forward SSH cDNA library to confirm if the ESTs population in the library indeed represented the genes overexpressed due to salt stress. It was also done to validate the EST redundancy in a functional category. For this, total RNA was isolated from the leaves of the control and NaCl-treated *S. maritima *as described above and 10 μg RNA per slot was vacuum blotted on to nylon membrane (N^+ ^Hybond, Amersham). The blots were air-dried and UV cross-linked at 150 mJ using a UV cross linker (GS Gene linker, Bio-Rad). These were hybridized with the probe made by random primer α^32^P-dATP labelling of the ESTs of interest. The PCR amplified actin fragment was radiolabelled similarly. This was hybridized with a RNA blot each of a control and a NaCl treated sample for the normalization of the RNA loading in the two cases. The hybridization and washing conditions were as described above [41].

In order to verify further the salt-induced expression or enhanced expression of the unigenes, real-time RT-PCR (qRT-PCR) was conducted for five of them encoding jasmonic acid induced protein, JAIP (FC932662), catalase (FC932734), phosphoethanolamine N-methyltransferase, PEAMT (FC932718), Δ^1^-pyrroline-5-carboxylate synthetase, P5CS(FC932725) and DnaJ (FC932656). The selected unigenes varied greatly in EST redundancy. qRT-PCR was also performed to check the influence of NaCl on the expression of the gene encoding betainealdehyde dehydrogenase (BADH), the ultimate gene in the pathway of glycinebetaine synthesis from choline in plant. The gene encoding actin was amplified simultaneously for each set of qRT-PCR reaction for comparison and normalization of the data. RNA was isolated as and when required from the leaves of the control and NaCl treated plants as described above and treated with DNase to remove any DNA contamination. The quality and quantity of the RNA in each preparation was checked spectrophotometrically using NanoPhotometer (Implen, GmbH). The qRT-PCR reaction was conducted using QuantiFast SYBR Green RT-PCR kit (Qiagen, USA) and Opticon-2 qRT-PCR machine (MJ Research, Bio-Rad). Each RT-PCR reaction mixture was prepared as per the instructions in the kit taking 100 ng of RNA and 1 μM gene-specific primer in a final volume of 25 μl. The primers for all the genes, except *BADH*, were designed based on the nucleotide sequence information of their ESTs. For designing the primer for *BADH*, the nucleotide sequence information of its full-length cDNA clone from *Suaeda salsa *[DQ641924] was considered. The primers were obtained from Gene Link (NY, USA). The primer sequences for the various genes were: *JAIP*-For5'CAATCAAAGCTCCCTTTTCG, Rev5'AAGCCCGAAAACTCCACTCT; *Cat*-For5'GAGTGGTTGATGCCCTGTCT, Rev5'TCTCATCTCGATCCCCAAAG; *PEAMT*-For5'TTGCCCTTGAGCGTTCTATT, Rev5'TACCTCCTGGCTTCAACCAT; *P5CS*-For5'GATGTTTTTGCTGCCATTGA, Rev5'GCTAATCCCAACCTCAGCAC; *DnaJ*-For5'GGAATACAGGAGGGGGACAT, Rev5'CCTTTTGGGAGAACCAAACA; *BADH*-For5'TGGAAAATTGCTCCAGCTCT, Rev5'CTGGACCTAATCCCGTCAAA; *Actin*-For5'AAACCACAAGCCCCTAAACC, Rev5'TTGCATCACTCAGCACCTTC. The PCR reaction conditions were also set as per the instruction manual in the kit. After the completion of the reactions, threshold cycle (*C*_*T*_) value for each reaction was obtained with the help of the software attached with the machine and the difference in the transcript level (in fold) between the control and NaCl treated sample was calculated using Pfaffl method [42] considering the *C*_*T *_value of actin as the internal control. The fold change in the transcript levels of each gene (considered for qRT-PCR) upon NaCl treatment was presented as the mean ± standard deviation (sd) of three independent experimental analysis.

### Enzyme activity study

The effect of NaCl on the activity of two enzymes Δ^1^-pyrroline-5-carboxylate synthetase (P5CS, EC 1.5.1.12) and catalase (Cat, EC 1.11.1.6) was studied. The leaves of the control and NaCl treated plants were homogenized separately in chilled enzyme extraction buffer (100 mM Tris-HCl, pH 7.8 containing 10 mM MgCl_2_, 1 mM PMSF, 0.1 mM EDTA, 2% PVPP, 1% protease inhibitor cocktail and 10 mM DTT) in a cold room using pre-chilled mortar and pestles [20,43]. The homogenates were centrifuged twice at 4°C for 20 min at 20000 × g. The protein in the supernatants was quantified by coomassie brilliant blue-dye binding method [44].

P5CS activity in the enzyme extract was determined as γ-glutamyl kinase by monitoring the formation of γ-glutamyl hydroxamate [45]. The enzyme mixture in a final volume of 0.5 ml contained 50 mM Tris-HCl (pH 7.0), 50 mM L-glutamate, 20 mM MgCl_2_, 100 mM hydroxamate-HCl, 10 mM ATP and 50 μl enzyme extract. After addition of the enzyme extract, the reaction mixture was incubated at 37°C for 15 min. The reaction was stopped by adding 1 ml of the stop buffer (2.5 g FeCl_3 _and 6 g trichloroacetic acid in a final volume of 100 ml of 2.5 M HCl). The precipitated proteins were removed by centrifugation, and the absorbance of the clear supernatant was read at 535 nm against a blank identical to the above but lacking ATP. The activity was expressed as the unit (U) mg^-1 ^protein; 1 U represented the amount of the enzyme (protein) required to produce 1 μmol of γ-glutamyl hydroxamate (molar extinction co-efficient- 250 M^-1 ^cm^-1^) in one min. The data presented are the means of at least three independent analyses.

The activity of catalase in the supernatant was measured following the method of Chance and Maehly [46] with some modification. The reaction mixture for catalase contained 25 mM potassium phosphate buffer (pH 6.8), 20 mM H_2_O_2 _and the enzyme extract. The reaction was started by adding the enzyme extract. The decomposition of H_2_O_2 _was followed at 240 nm, and was quantified using a standard graph prepared for H_2_O_2 _concentration. The activity was expressed as U mg^-1 ^protein, where 1 U is the amount of the enzyme (protein) required to decompose 1 μmol of H_2_O_2 _in 1 min. The data presented are the means of at least three independent analyses.

The significance of difference in the enzyme activity between the samples was checked by Duncan's multiple range test for unequal sample size [47].

### In-gel catalase activity study

The effect of NaCl on the activity of catalase was also studied by in-gel activity staining of the enzyme activity. The enzyme extracts from the leaves of the control and NaCl treated plants were obtained as above. The homogenizing buffer contained 10% glycerol in addition to the other ingredients [48]. The individual supernatant was mixed with 3× loading buffer (190 mM Tris HCl, pH 6.8, 20% glycerol, 65 mM DTT, 0.002% bromophenol blue) in 2:1 ratio and loaded on to a native gel (7. 5% separating and 4% stacking) supported by 10% glycerol [48]. Equal amount of protein (40 μg) was loaded in each lane and the electrophoresis was conducted in a cold room by applying 10 mA current for the stacking gel and 20 mA for the separating gel. The electrophoresis was allowed to continue for 2 h after the dye crossed the separating gel. The gel was removed, soaked in 3.27 mM H_2_O_2 _for 25 min, rinsed quickly with distilled water and stained with solution containing 1% (w/v) potassium ferricyanide and 1% (w/v) ferric chloride. The presence of catalase was visualized as negative band. The progress of staining was stopped by removing the staining solution and adding 1% HCl.

## Results

### SSH library construction and their differential screening

**Figure 1:**
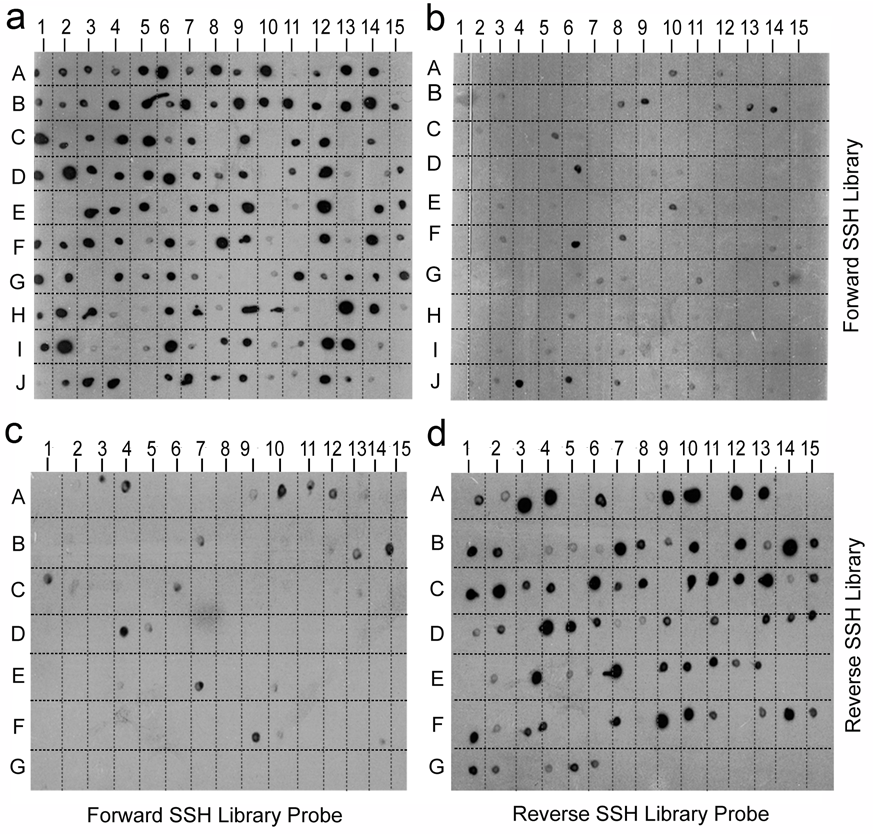
**Results of differential screening of the clones from forward and reverse SSH cDNA libraries**. Young *S. maritima *plants were exposed to 425 mM NaCl for 24 h (treated). The plants of the same age not receiving NaCl treatment served as control. Plates a and b: the membranes were blotted with clones from the forward SSH cDNA library. Plates c and d: the membranes were blotted with the clones from the reverse SSH cDNA library. Plates a and c: the blotted membranes were screened by the probe made from the forward subtracted SSH cDNA. Plates b and d: the blotted membranes were screened by the probe made from the reverse subtracted SSH cDNA.

The agarose plating of the competent *E. coli *cells transformed for the ESTs from the forward and the reverse SSH cDNA libraries yielded several transformed colonies. From the four plating done for each SSH cDNA library, 492 recombinant colonies for the forward and 502 colonies for the reverse library could be picked-up. The results of the differential screening of the EST clones from both the forward and the reverse SSH cDNA libraries are shown in Fig. 1. Most of the 150 spotted plasmids from the randomly picked transformed colonies generated for the forward subtracted SSH cDNA showed hybridization signal with the probe made from the secondary PCR product of the forward subtracted SSH cDNA (Fig. 1a). The intensity of the spots varied greatly suggesting the presence of variable number of transcript messages of the individual overexpressing genes. Upon hybridization of the duplicate blot with the probe made from the secondary PCR product of the reverse subtracted SSH cDNA, only a few hybridization signals were observed (Fig. 1b). This suggested that the transcript messages present in the forward SSH cDNA library were different from that present in the reverse SSH cDNA library, and that the library represented mostly the salt-induced transcript messages. A few hybridization signals obtained could be an artefact or the subtraction of the cDNAs of a few overexpressing genes might not have been total during the preparation of the reverse SSH cDNA library.

For the screening of the reverse SSH cDNA library, 96 plasmid DNA samples, isolated from the randomly picked transformed bacterial colonies obtained for the reverse subtracted SSH cDNA, were blotted and hybridized with the probe made from the secondary PCR product of either the reverse or the forward subtracted SSH cDNA. As expected, most of the 96 spots gave hybridization signal with the probe made from the secondary product of the reverse subtracted SSH cDNA (Fig. 1d), but only a few hybridization signals were observed with the probe made from the secondary PCR product of the forward subtracted SSH cDNA (Fig. 1c). This suggested that the removal of the salt-induced or -unaffected messages during the subtraction step was more or less complete while constructing the reverse SSH cDNA library, and that the library represented mostly the messages that were down regulated due to the salt stress.

### Sequencing of the forward subtracted SSH cDNA, contig assembly and annotation

The cloned ESTs of only the forward SSH cDNA library were considered for sequencing. This is because these represented the genes overexpressing in response to the NaCl treatment, and hence could be more relevant from the point of view of salt tolerance than the genes down-regulated by the NaCl treatment, represented by the reverse SSH cDNA library. Only 429 clones were found to be good for annotation and contig assembly (Table 1). These ESTs could be grouped into 89 singletons and 78 contigs represented by 340 ESTs with an overall EST redundancy of 81.8% (Table 1). Thus, the forward SSH cDNA library represented 167 unigenes (the combined set of contigs and singletons), which either overexpressed in response to the NaCl treatment or expressed only after the NaCl treatment. More than half of the ESTs from the forward SSH cDNA library could be assigned putative function on the basis of the sequence similarity to the genes or proteins of known function in the GenBank (see Additional file 1). The maximum similarity of the ESTs to a given protein in the database in terms of BLASTX E value is also given in the table. More than 30% of the unigenes showed no match in the protein database, and approximately 4% of the sequences represented proteins whose function is not known (see Additional file 1). Most of the unigenes, which represented known proteins showed BLASTX E value < 10^-2^. Only 15 unigenes finding matches in the protein database showed BLASTX homology at E > 10^-2^, and of these nine were either hypothetical/unknown proteins or proteins with putative function. Hence, these may be considered as the unigenes with no match in the database. All the EST sequences are available at NCBI [GenBank: FC932656–FC932657, FC932659–FC932666, FC932668–FC932807 and FG228208–FG228224]

**Table 1:** ESTs summary of the forward SSH cDNA library of *S. maritima*.

**Descriptive category**	**Values**
No. of high quality ESTs	429
Mean EST length (bp)	392
EST size range (bp)	74–814
No. of singletons	89
No. of contiguous sequences (contigs)	78
No. of unigenes	167
No. of ESTs in contigs	340
Contig EST redundancy (%)^a^	81.8
Maximum EST redundancy in a contig (%)^b^	7.7

Forward SSH cDNA library, representing the salt-induced genes, was constructed considering the mRNA isolated from the leaves of the NaCl-treated (24 h) plant as 'Tester' and that from the leaves of the control plant as 'Driver'. The cDNAs of the library were cloned and transformed and 502 ESTs from such clones were sequenced. The results are summarized.

^a^Percentage of the faction of ESTs assembled in the contigs/total no. of ESTs.

^b^Percentage of the fraction of ESTs in a contig/total no. of ESTs

Among the unigenes identified, that reported to be induced by jasmonic acid showed the highest expression; the EST redundancy of this particular gene was found to be as high as 7.69% (Table 2) out of the total contig EST redundancy of 81.8% (Table 1). In fact, two isoforms of the gene encoding jasmonic acid-induced protein (JAIP) were found to be overexpressing in the test plant upon NaCl treatment, one with EST redundancy of 2.80% and the other with EST redundancy of 7.69%. Down the line, the next gene showing high EST redundancy was that encoding homeodomain leucine zipper transcription factors, ATB-1 (Homeobox leucine zipper) and HDZ3 (Homeodomain leucine zipper), each showing EST redundancy of 3.76%. The transcription factor EREBP (Ethylene responsive element binding protein) and a putative zinc binding protein with RING domain (Zn-finger protein, ZnF) were among the protein products of highly overexpressing (NaCl-induced) genes after *ATB-1 *and *HDZ3 *showing EST redundancy of 0.89 and 1.17%, respectively. In addition, there was overexpression of genes of two other transcription factors, C2H2 zinc finger (C2H2-ZnF) family protein and white collar (WC1) protein, and also of a protein, pasticcino-1 (PAS1) involved in regulation of the NAC transcription factors. The EST redundancy of these genes were, however, very low.

**Table 2:** Unigene sequences (ESTs) representing proteins with regulatory roles.

**EST accession number (GenBank)**	**Name of the proteins/genes**	**EST redun-dancy for a gene (%)^a^**	**BLASTX search E-value**	**Mean EST length (bp)**
FC932788	60S acidic ribosomal protein P0	0.93	4.00E-46	657
FG228222	Adenosine monophosphate binding protein-5 (AMP-binding)	0.23	1.00E-64	543
FC932702	Appr-1-p processing enzyme family protein	1.40	8.00E-12	132
FC932664	ATHB-1 (Homeobox-leucine zipper protein)	3.76	4.00E-18	373
FC932731	C2 domain-containing protein	0.23	3.00E-14	601
FC932705	C2H2 type zinc finger family protein	0.23	3.00E-09	326
FC932694	C3H4-type zinc finger (RING finger) protein	0.23	2.00E-12	247
FC932783	CBS domain-containing protein	0.23	2.00E-25	254
FC932656	DnaJ protein, putative^b^	0.47	4.00E-67	467
FC932677	Ethylene responsive element binding protein (EREBP)	0.93	6.00E-16	160
FG228218	Eukaryotic elongation factor 1A	0.23	3.00E-42	321
FC932688	F-box domain containing protein, putative	0.23	0.056	509
FC932771	GTP-binding protein, putative	0.23	8.00E-44	276
FC932675	Homeodomain leucine zipper protein HDZ3^b^	3.76	2.00E-18	373
FC932662	Jasmonate-induced protein homolog	7.69	0.001	386
FC932679	Jasmonate-induced protein homolog	2.80	1.00E-05	520
FC932804	Leucine-rich repeat family protein	0.23	9.00E-28	315
FC932737	O-linked GlcNAc transferase like	0.93	1.00E-04	307
FC932759	Pasticcino 1	0.23	1.00E-62	626
FC932684	Pre-mRNA splicing factor ATP-dependent RNA helicase-like protein^b^	0.23	2.30	610
FC932706	Putative sigma-like transcription factor	0.23	2.00E-09	271
FC932765	Putative Zn-binding protein with RING finger	1.17	7.00E-22	457
FG228209	Transducin family protein	0.23	1.00E-47	380
FG228219	Valyl-tRNA synthetase, putative	0.23	6.00E-36	539
FC932666	White collar 1 protein (WC1)	0.23	9.70	311

ESTs sequences from the forward SSH cDNA library of *S. maritima *were grouped into singletons and contigs using TIGR Assembler and were termed as unigenes. The unigene sequences were blasted for homology search using BLASTX programme (default) at NCBI database. The search results for those unigenes representing proteins having regulatory function are summarized. EST redundancy of each unigene is also given along with the average size of the ESTs constituting the unigene.

^a^Percentage of the fraction of ESTs representing a unigene/total no. of ESTs

^b^Genes reported to be induced by salt

Besides that of transcription factors, the expression of genes encoding several other proteins with regulatory function was also found to be enhanced in the plant in response to the NaCl treatment (Table 2). One group among them consisted of the genes encoding proteins with various recognized domains, such as CBS, F-box and C2, and motifs such as C3H4 zinc finger and leucine rich repeat, mediating protein-protein interaction in various biochemical events such as polyubiquitination, transcription elongation, centromeric binding, translational elongation, membrane trafficking, etc. The second group was comprised of the genes of G protein (Transducin, GTP binding protein) and AMP-binding (Adenosine monophosphate binding) protein, which are involved in signal perception and transduction. The genes of other proteins with some possible regulatory role that overexpressed in response to the NaCl treatment was O-linked GlcNAc (N-acetylglucosamine) transferase (OGT) regulating protein function by O-linked β-N-acetylglucosamine addition on the serine/threonine residue, and DnaJ like protein functioning as co-chaperones helping in protein translation, translocation, folding, assembly and deassembly. Besides, the expression of the genes encoding proteins constituting the protein synthesis machinery itself, like preRNA splicing factor, sigma like transcription factor, 60S ribosomal P0 protein, appr-1p (ADP-ribose 1"-phosphate) processing enzyme family protein, eukaryotic elongation factor 1A and valyl tRNA synthetase involved in transcription, mRNA and tRNA processing and translation was greatly increased in response to the salt treatment. The most significant enhancement in the expression among them was of the gene encoding 60S acidic ribosomal P0 showing EST redundancy of 0.93%.

A clear distinguishing feature of differential gene expression in the test plant in response to the NaCl treatment was the overexpression of the genes encoding proteins performing various physiological functions related to adaptation of plants to saline and/or drought conditions (Table 3). At least three of these gene products function in association with the cellular membranes. The NaCl-induced expression of the gene encoding one among them, the choline transporter, was the maximum in the group; two isoforms were found to be expressing with a combined EST redundancy of 2.82%. The second gene encoding the membrane associated protein with high EST redundancy was the cation-efflux transporter; overexpression of two isoforms of the gene was observed in this case as well. A putative Na^+^/H^+ ^antiporter was the third gene that was found to be overexpressed under NaCl stress, although the BLASTX sequence homology for the gene product was very less (E = 7.6). Besides the genes encoding membrane proteins, the genes for the enzymes possibly playing important role in cell wall formation, for example xyloglucan endotransglycosylase (2 isoforms) and expansin-3, also showed overexpression with high EST redundancy.

**Table 3:** The Unigene sequences (ESTs) representing proteins important for the salt adaptive phsiological processes.

**EST accession number (GenBank)**	**Name of the proteins/genes**	**EST redun-dancy for a gene (%)^a^**	**BLASTX search E-value**	**Mean EST length (bp)**
FC932725	Δ^1^-pyrroline-5-carboxylate synthetase^b^	0.23	5.00E-36	227
FC932672	Carbonic anhydrase^b^	0.70	2.00E-73	606
FC932674	Carbonic anhydrase^b^	0.70	5.00E-73	574
FC932784	Cation-efflux transporter	0.93	9.00E-66	686
FG228211	Cation-efflux transporter	0.23	5.00E-64	581
FC932758	CCL (CCR-LIKE) protein	0.93	0.24	152
FC932738	Choline transporter-related^b^	0.93	3.00E-14	295
FC932763	Choline transporter-related^b^	1.89	1.00E-16	305
FC932670	Expansin 3	0.47	3.00E-23	727
FC932700	Methionine adenosyltransferase	0.93	2.00E-74	407
FC932718	Phosphoethanolamine N-methyltransferase^b^	0.70	8.00E-124	814
FC932801	Phosphoethanolamine N-methyltransferase^b^	0.70	1.00E-140	803
FG228215	Phosphoethanolamine N-methyltransferase^b^	0.23	2.00E-66	804
FC932680	Putative Na(+)/H(+) anti-porter	0.47	7.6	225
FC932696	S-adenosyl-L-homocystein hydrolase	0.23	2.00E-74	486
FC932721	Xyloglucan endotransglycosylase 1	0.70	1.00E-31	441
FC932774	Xyloglucan endotransglycosylase 1	0.70	5.00E-30	439

BLASTX results for those unigenes representing proteins performing various physiological functions related to adaptation of plants to saline condition and possibly vital for the salt adaptive physiological processes. Other details as in Table 2.

^a^Percentage of the fraction of ESTs representing a unigene/total no. of ESTs

^b^Genes reported to be induced by salt

**Figure 2:**
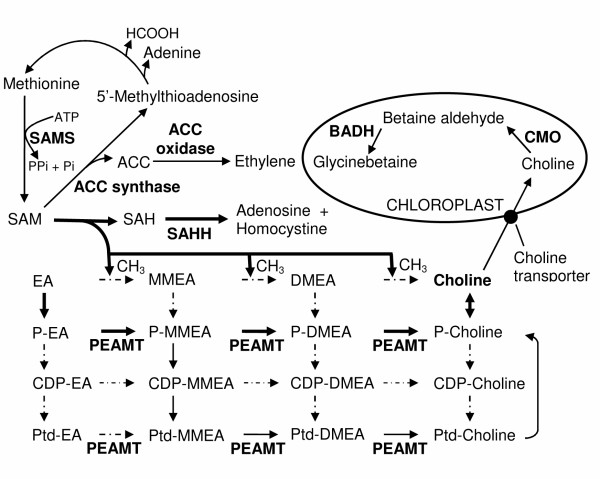
**Biosynthetic pathways of glycinebetaine and ethylene in plant**. Glycinebetaine in plant is synthesized from choline, which in turn is synthesized by three sequential N-methylation of NH_2_-moiety of ethanolamine (EA), phospho-ethanolamine (P-EA) or phosphatidyl-ethanolamine (Ptd-EA) catalysed by phosphoethanolamine N-methyltransferase (PEAMT). Monomethylehthanolamine (MMEA), dimethylethanolamine (DMEA) and their phsopho- (P-), citidine diphosphate- (CDP-) and phosphatidyl- (Ptd-) derivatives are various intermediates. Methyl group is donated at each step by SAM (S-adnosylmethionine). SAM is also the methyl donor for ethylene synthesis catalysed by ACC synthase and ACC oxidase. The by-product SAH is broken down further by SAHH (S-adenosylhomocysteine hydrolase). SAM is regenerated from methionine by SAMS (S-adenosyl-L-methionine synthase).

Overexpression of the genes known to be directly or indirectly related to a well established physiological adaptation process in plants to salt or drought stress, the osmotic adjustment, was prominently reflected in the test plant in response to the NaCl treatment (Table 3). The most overexpressing gene in this category was that encoding phosphoethanolamine N-methyltransferase (PEAMT) related to the synthesis and accumulation of glycinebetaine, a well known compatible solute for osmotic adjustment in plants under salt and drought stresses. The enzyme catalyzes the conversion of phosphoethanolamine (P-EA) to phosphocholine, a precursor of choline and glycinebetaine (Fig. 2). Three isoforms of *PEAMT *were detected with a combined EST redundancy of 1.63%. Besides, the expression of the gene encoding methionineadenosyl transferase (S-adenosyl-L-methionine synthetase, SAMS), the enzyme responsible for the synthesis of S-adenosylmethione (SAM) required for the conversion of ethanolamine (EA) to P-EA by methylation (Fig. 2), also increased greatly showing EST redundancy of 0.93%. During the transmethylation reaction, SAM is converted to S-adenosyl-L-homocysteine (SAH), which is inhibitory to all SAM dependent methyltransferases, and hence it should be metabolized and recycled, which is done by SAH hydrolase, SAHH (Fig. 2). The expression of *SAHH *was also enhanced in the plant by the salt treatment. In addition to the overexpression of the genes encoding the enzymes involved in glycinebetaine synthesis, the study also revealed enhanced expression of the gene encoding Δ^1^-pyrroline-5-carboxylate synthetase (P5CS), an enzyme involved in the biosynthesis of proline, which is another well known osmoticum.

Among the other genes highly overexpressing and possibly having important role to play in the physiological processes leading to adaptation of a plant to abiotic stress were those encoding CCR (Cold-circadian rhythm-RNA binding)-like (CCL) protein and two isoforms of carbonic anhydrase (CA); while *CCL *expressed with an EST redundancy of 0.93%, the two isoforms of *CA *expressed with a combined EST redundancy of 1.40% (Table 3). The genes of the other known enzymes and proteins performing important metabolic functions, which were found to be overexpressed, although with low EST redundancy, are listed in the Supplementary table (see Additional file 1)

### Northern blot and qRT-PCR analysis of representative ESTs

**Figure 3:**
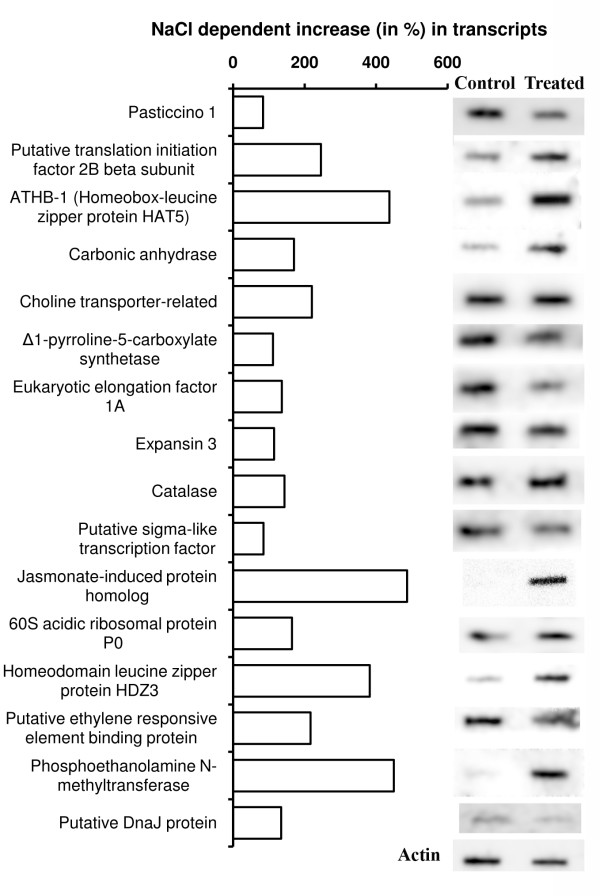
**Northern analyses of a few select forward subtracted SSH cDNA clones**. RNA isolated from the leaves of the control and 425 mM NaCl-treated plants and blotted onto Hybond N^+ ^membrane was hybridized with the individual radiolabelled ESTs. A RNA blot each for the control and NaCl-treated sample was hybridized with PCR amplified radiolabelled actin fragment. The horizontal bars against the individual genes represent increase (in%) in transcripts of the respective genes in response to NaCl treatment of the plant when compared to control. The values were obtained after normalization of the blot intensities of actin for the control and NaCl treated sample.

**Figure 4:**
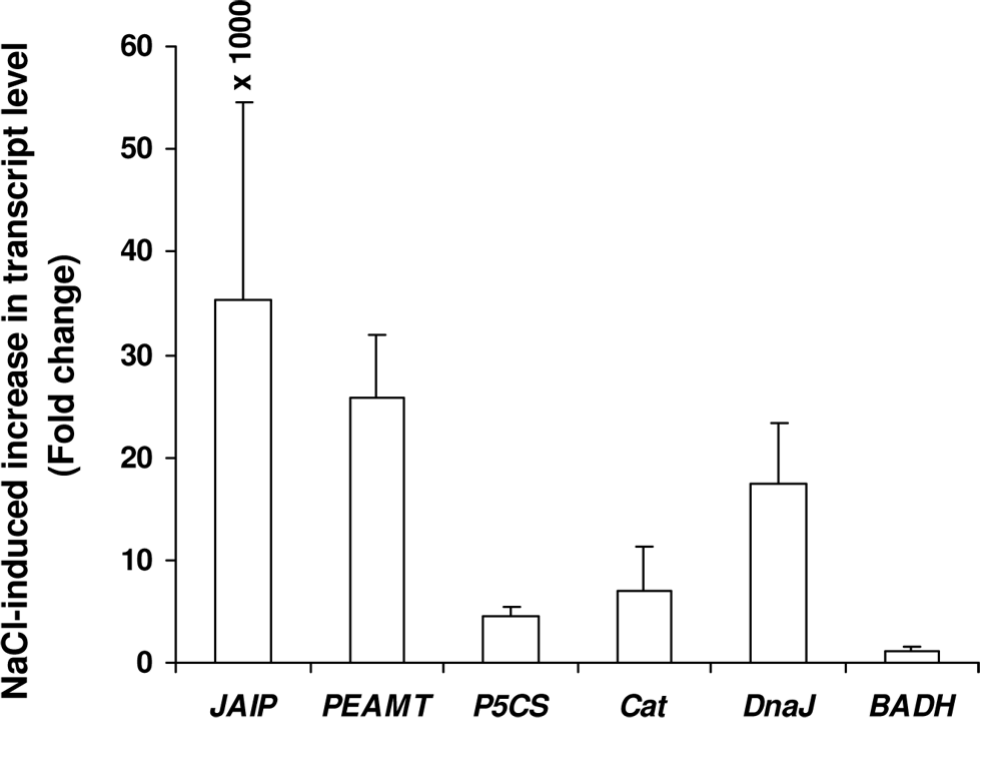
**Expression study by Real-time RT-PCR of a few unigenes varying in EST redundancy**. Real-time PCR (qRT-PCR) was conducted for five unigenes encoding JAIP, catalase, PEAMT, P5CS and DnaJ to see the effect of NaCl treatment on their expression. qRT-PCR was also performed for *BADH*. Actin gene served as the internal control. RNA isolated from the leaves of control and NaCl treated plants was individually set for qRT-PCR using QuantiFast SYBR Green RT-PCR kit (Qiagen, USA) and 1 μM gene-specific primer. Each bar represents the number of fold increase in the transcript level of a gene in the plant upon NaCl treatment compared to the control level. The values presented are the mean ± standard deviation (sd) of three independent experimental analysis.

To validate further that the EST population of the forward SSH cDNA library really represented the population of the genes that overexpressed or additionally expressed in the test plant upon the salt treatment, Northern blot analysis was performed for a few select cDNA clones that varied in EST redundancy (Fig. 3). Overexpression of all these genes under salt stress was visible. Hybridization signal for the gene encoding JAIP was not detected in the control plant, suggesting that the gene was expressed only upon exposure of the plant to NaCl. Varying probe hybridization signal was observed for the genes encoding different proteins, and when the signals were normalized with the respective actin signal, the level of expression of the individual genes quite matched their EST abundance (or redundancy) in the forward SSH cDNA library. The qRT-PCR data also revealed very high expression of the gene encoding JAIP (Fig. 4). Overexpression of *P5CS *in the NaCl-treated plant was the least among the genes selected for qRT-PCR. This was also reflected in the slot-blot hybridization (Fig. 3). However, the qRT-PCR result showed a greater overexpression of *DnaJ *in the NaCl treated plant when compared to the result of the slot-blot hybridization. A slight overexpression of *BADH *was detected in the test plant upon NaCl treatment, although cloning and sequencing of the cDNAs from the forward subtracted SSH cDNA library did not show any presence of EST of the gene.

### Activity assay of catalase and P5CS

**Figure 5:**
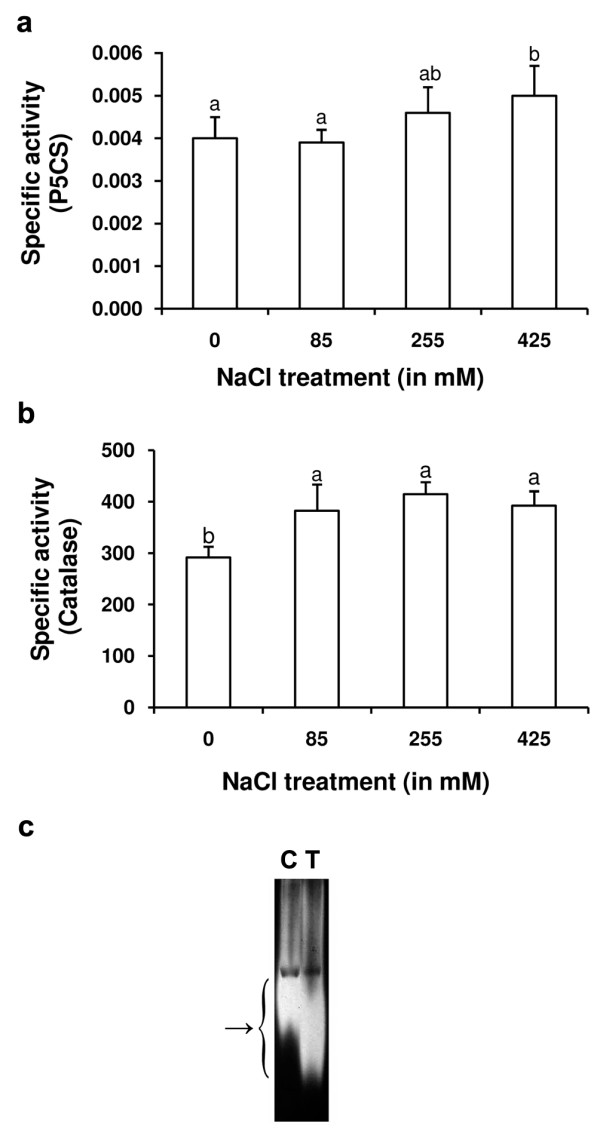
**Activity study of Δ^1^-pyrroline-5-carboxylate synthetase (P5CS) and catalase**. The enzyme extract from the leaves of control and NaCl treated plants was assayed for the activity of P5CS (a) and catalase (b). The individual columns represent the mean specific activity (Unit mg^-1 ^protein) value of at least 3 replicate studies. The vertical bars represent standard deviation. The mean specific activity values (columns) of P5CS or catalase marked with at least one common alphabet are not different significantly from each other at p ≤ 0.05, as found by Duncan's multiple range test for unequal sample size. Catalase extracted from the leaves of the control (C) and 425 mM NaCl treated (T) plants was loaded and run on native gel and stained for in-gel activity (c). The staining shows noticeable increase in the activity of the enzyme in response to the NaCl treatment.

The activity of catalase and P5CS was checked to validate the result of the forward subtractive hybridization at the physiological level. The genes of these two enzymes overexpressed with the least EST redundancy (Table 2). The activity of P5CS showed significant increase only at 425 mM NaCl treatment (Fig. 5a). Catalase on the other hand showed significant increase in its activity in the plant at all the NaCl-treatment concentrations (Fig. 5b). The increase in the activity of the enzyme was, however, not NaCl-concentration dependent, as the increase was similar at all the treatment concentrations. The accelerating effect of NaCl on the activity of catalase is also visible from the in-gel assay of the enzyme activity performed for the plant exposed to 425 mM NaCl treatment concentration (Fig. 5c).

### Functional categorization of the unigenes

**Figure 6:**
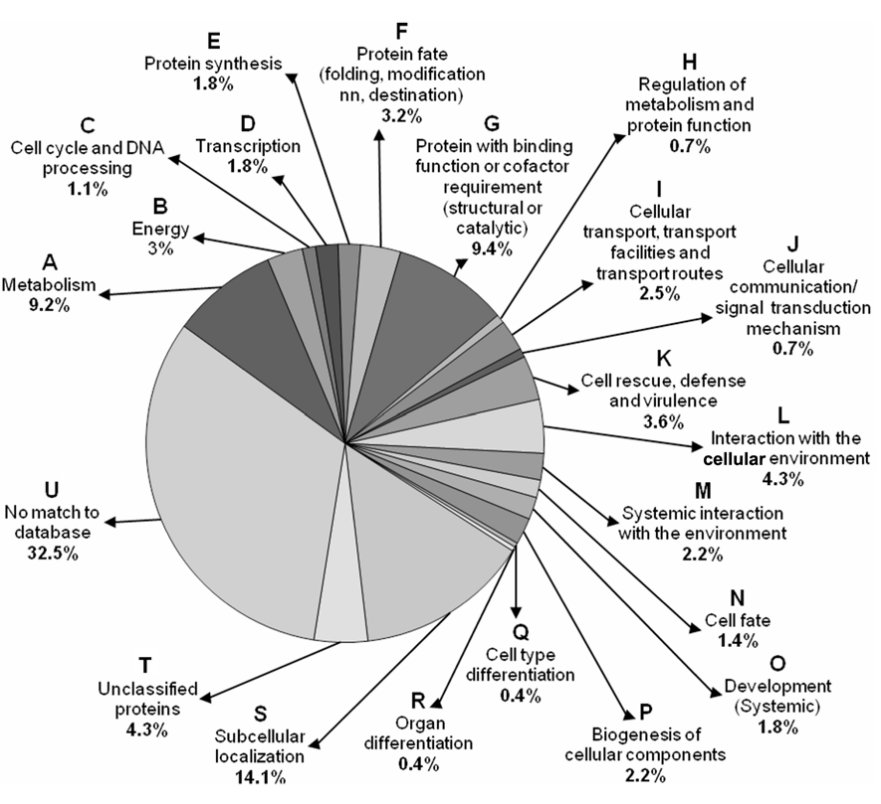
**Functional categorization of the unigenes**. The unigenes (167 numbers) found to be overexpressing in response to salt treatment in the present study were grouped into 19 functional sub-categories (A-S) under 6 main functional categories, namely metabolism (sub-category A-B), information pathways (sub-categories C-H), transport (sub-category I), perception and response to stimuli (sub-categories J-M), developmental processes (sub-categories N-R) and subcellular localization (sub-category S). Experimentally uncharacterized proteins were put into the sub-category T (representing unclassified/unnamed proteins) and U (representing protein with no match in the database). The percentage of the total unigenes representing a sub-category is given against it.

The functional classification of the unigenes was carried out using MIPS (Munich Information Centre for Protein Sequences) functional catalogue , which is based on the pathways where the proteins act. These were classified into 19 functional sub-categories (marked A-S, Fig. 6) excluding the unclassified (unknown and unnamed) proteins (marked T, Fig. 6), representing 4.3% of the unigenes, and the proteins for which no matches were found in the database (marked U, Fig. 6). The latter represented nearly one third (32.5%) of the unigenes. Among the functional categories, the genes encoding proteins responsible for subcellular localization of biomolecules (sub-category S) were found to be expressing the most making 14.1% contribution. The genes encoding protein with binding function or co-factor requirement (sub-category G) followed the next with a contribution of 9.4%. In fact, this sub-category is a constituent of a major functional category, the information pathways (Fig. 6, sub-categories C-H). This represented 18% of the total unigenes belonging to the various other sub-categories like cell cycle and DNA processing (1.1%, C), transcription (1.8%, D), protein synthesis (1.8%, E), protein fate (3.2%, F) and protein activity regulation (0.7%, H), in addition to the sub-category G (Fig. 6). The next highest representation was by the genes encoding proteins involved in metabolism (Fig. 6, sub-categories A and B), contributing 11.6% of the total unigenes. The genes encoding proteins concerned with perception and response to stimuli (Fig. 6, represented by sub-categories J-M), such as those involved in cellular communication (J, 0.7%), cell rescue (K, 3.6%), interaction with the cellular environment (L, 4.3%) and systemic interaction with the environment (M, 2.2%) constituted 10.8% of the total unigenes. The representation by the genes encoding proteins taking part in developmental processes, such as cell fate (sub-category N, 1.4%), systemic development (sub-category O, 1.8%), biogenesis of cellular components (sub-category P, 2.2%), cell type differentiation (sub-category Q, 0.4%) and organ differentiation (sub-category R, 0.4%) was found to be the least (6.2%) in the total unigenes.

**Figure 7:**
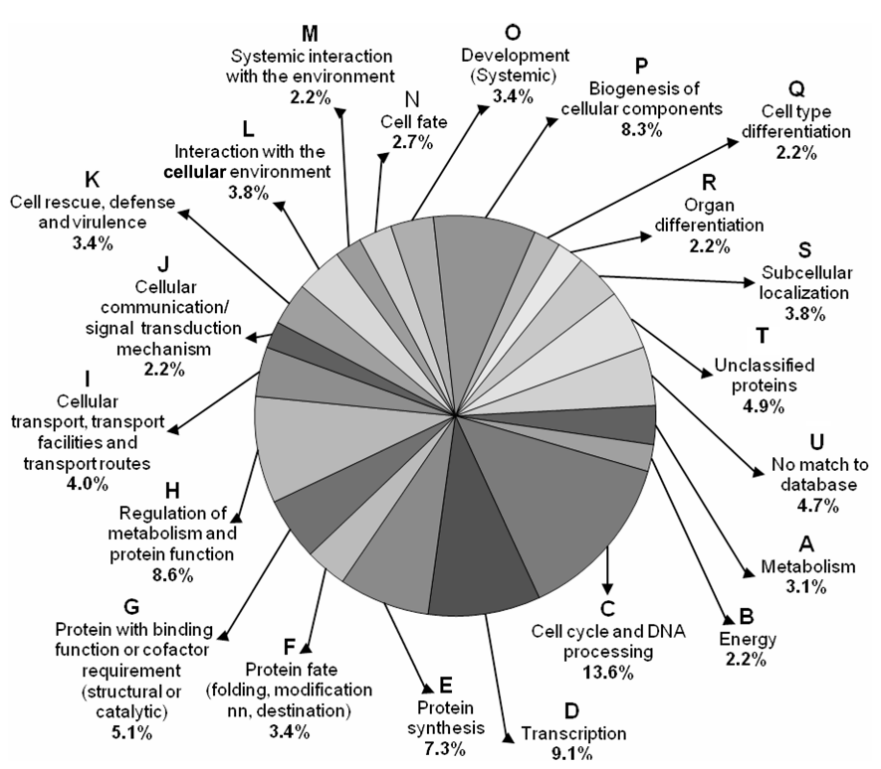
**ESTs redundancy in individual functional categories/sub-categories**. Functional categorization of the unigenes is same as in Fig. 6. The percentage value against each sub-category represents EST redundancy (in%) in that particular group, which was calculated as the percentage of fraction of the number of ESTs representing the group/total number of ESTs.

Representation of the ESTs in the information pathway (Fig. 7, sub-categories C-H) was the highest (47.1%). The second highest EST representation (18.8%) was for the proteins involved in developmental processes (Fig. 7, sub-categories N-R), although the total unigene representation in this category was the least (6.2%). The genes encoding proteins involved in perception and response to stimuli (Fig. 7, sub-categories J-M) also made a greater EST representation (11. 6%) than the representation made by the total unigenes (8.6%) in the group. The relative overexpression of the genes encoding protein involved in subcellular localization (Fig. 7, sub-category S) was the least (3.8%). While the percentage of EST representing the unclassified proteins (Fig. 7, sub-category T) remained similar to that of the total unigene representation in the group, the EST representation for the proteins with no similarity in the database (Fig. 7, sub-category U) was found to be very less (4.7%) compared to the representation made by the unigenes (32.5%) in the group (Fig. 6).

## Discussion

Over the last decade, vast insight into the plant growth and development and other plant processes have been gained because of the growth and development in molecular biology techniques. Suppression subtractive hybridization (SSH) is among such techniques being largely used to isolate the genes that are differentially expressed in contrasting environments. Although the PCR-based SSH technique has been used to know the genes differentially expressed in some plants under salt stress [18,19,40,49], no report exists on the salt-responsive genes in natural halophyte, which is likely to give better information on the genes relevant to salt tolerance than the studies carried out on other plants. The present study has been an attempt in this direction. Although SSH is a powerful technique that enriches the differentially expressed genes, it is by no means perfect. This is also evident from the present study as the probe prepared using the reverse subtracted SSH cDNA showed hybridization with the forward subtracted SSH cDNA clones, or *vice-versa *(Fig. 1b, c), which was not expected. Northern blot analysis of select forward subtracted cDNA clones, including that showing hybridization with the radiolabelled reverse subtracted SSH cDNA, nevertheless, revealed that these clones (ESTs) actually represented the genes overexpressing in response to the NaCl treatment (Fig. 3). The Northern blot hybridization also revealed that the SSH process was in fact dependent on the expression level of a gene, as the genes showing high ESTs redundancy showed greater hybridization signal than those showing low ESTs redundancy (Fig. 3, Table 2). Expression analysis of a few genes by qRT-PCR also confirmed the same; the NaCl-induced changes in the transcript levels of the selected genes (Fig. 4) quite paralleled their ESTs redundancy (Table 2 and 3). Besides, the qRT-PCR analysis also revealed that although the ESTs of the genes like *P5CS *and *Cat *were present in very low redundancy, their expression in response to the NaCl treatment had in fact increased by more than four fold. Hence, the genes showing low overexpression in response to a stress application may not find representation in the forward SSH cDNA library. It is also possible that the cDNAs of certain gene, present in high amount in both the 'Driver' and the 'Tester' cDNA population, may not be subtracted properly from the 'Tester' cDNA population in two rounds of hybridization with the 'Driver' cDNA, and thus the gene may find representation in the forward SSH cDNA library without actually overexpressing in response to a stress application [19].

The overexpression of as many as 167 unigenes while suggested involvement of a large number of genes in the salt tolerance process, contig EST redundancy of 81.8% indicated the possibility of discovery of more such genes, particularly that of the low abundance proteins, on continued cloning and sequencing of the forward SSH cDNA library. Moreover, more than 30% of the unigenes were novel, not reported before, and in addition approximately 4% were found to be producing proteins of unknown function (Table 1, Fig. 6, 7). These transcripts might represent important genes specific to salt tolerance. Although the EST redundancy of the genes in these groups is not high (Fig. 7), their importance in salt tolerance processes cannot be ignored (Fig. 7). The nucleotide and the corresponding amino-acid sequence data revealed that several clones isolated in this study, marked '^b^' in Table 2 and 3, and in the supplementary table (see Additional file 1), were significantly homologous to the salt stress-regulated genes/proteins reported for various plant species. Other proteins are not reported to be salt-induced.

The PCR-SSH has revealed overexpression of as many as twenty four transcription factors in tomato in response to NaCl-stress [18]. Using the same technique, Sahi *et al. *[19], however, has reported differential expression of genes of only two transcription factors, EREBP (ethylene responsive element binding protein) and Zn-finger (ZnF) protein, in two rice varieties in response to salt treatment. Our experiment on the other hand shows overexpression of the genes of six transcription factors in the test plant in response to the NaCl treatment (Table 2). The variation obtained could be species dependent. Nonetheless, the overexpression of the genes encoding EREBP and ZnF transcription factors are common among the three studies, suggesting important role of these proteins in the salt tolerance processes. EREBPs, currently known as ERE binding factor (ERF) proteins [50] belongs to a family of plant specific transcription factors characterized by the presence of ~60 amino acid highly conserved ERF/AP2 (APETLA2) DNA binding domain. A number of genes, including those encoding pathogenesis-related and antifungal proteins, are induced by various forms of biotic and abiotic stresses, such as pathogen attack, wounding, UV radiation, high or low temperature, drought and NaCl [50,51] mediated by ethylene produced in response to these stresses [51]. In addition, many of these have been found to contain ethylene responsive element (ERE), a cis acting element identified as GCC box for the interaction with ERF [52]. Certain *Arabidopsis *ERFs have also been reported to be induced by abiotic stresses, such as salinity, independent of ethylene signal transduction [50]. Enhanced expression of S-adenosyl-L-methionine synthase (*SAMS*) in the present case although indicated ethylene synthesis (Fig. 2) and ethylene dependent accumulation of ERF protein, the accumulation of ERF protein was most likely independent of the ethylene signal transduction. This is because there occurred no enhancement in the expression of ACC synthase (S-adenosyl-L-methionine methylthioadenosine-lyase) gene required for the conversion of S-adenosylmethionine to ACC (1-aminocycloropane-1-carboxylic acid), a rate limiting step in ethylene synthesis [53]. The increase in SAMS could be required to take care of the requirement of S-adenosylmethionine (SAM) for other biochemical reactions as the compound is the major methyl donor in plants and is used as a substrate for many biochemical pathways [54], involved in methylation reactions that modify lipids, proteins, and nucleic acids [53].

As for ethylene, no genes were identified in the forward SSH cDNA library that could be involved in the synthesis of jasmonic acid (JA) or methyl jasmonate (MeJA) from linolenic acid [55]. This is despite the fact that the forward SSH cDNA library showed the presence of ESTs for two isoforms of the gene encoding JAIP with a combined redundancy of as high as 10.49% (Table 1, Fig. 3, 4). Besides being induced by JA, a few JAIPs have also been reported to be induced by drought and salt [56,57]. The promoter of none of the genes encoding JAIPs has so far been studied. However, a jasmonate (and elicitor) responsive cis element (JERE) containing a GCC motif has been identified in the terpenoid involved alkaloid (TIA) biosynthetic gene strictosidine synthase, *Str *[58], which is recognised by a AP2 domain containing transcription factor ORCA2 (Octadecanoid-responsive *Catharanthes *AP2), similar to ERF, but its synthesis is induced by MeJa as elicitor instead of ethylene [58]. The involvement of the AP2-domain family members in both ethylene and JA signalling suggest that ethylene and JA may crosstalk via these transcription factors. Moreover, recently transcription factors like JERF1 (Jasmonate and ethylene response factor 1) and Tsil1 (Tobacco stress-induced gene 1) induced by NaCl, ethylene and JA have been discovered [49,59]. Besides binding to GCC box, these transcription factors also bind to dehydration responsive element (DRE)/C-repeat (CRT) involved in drought, salt and cold stress responses [60]. This expands the horizon of the crosstalk not only between ethylene and JA, but also among the other abiotic stresses, dependent or independent on ethylene/JA signalling for biological response.

The homeodomain zipper (HDZip) genes, *ATB1 *and *HDZ3*, were among the highest expressed transcription factors, which is not only reflected from their ESTs redundancy (Table 2), but also from the Northern blot result (Fig. 3). The combination of a homeodomain and a leucine zipper motif is unique to plant kingdom, suggesting that the HDZip genes may be involved in regulation of developmental processes specific to plants [61]. The functional information available on HDZip genes suggest that at least some of these genes are involved in mediating the effect of external conditions to regulate plant growth and development [62]. Several *A. thaliana *HDZip genes, like *ATHB-6*, -*7 *and -*12 *have been reported to be involved in abscisic acid (ABA) related response, including water deficit [63,64]. Several others, like *ATHB-7*, -*12*, -*6*, -*21*, -*40 *and -*53*, have also been reported to be overexpressed upon NaCl treatment, besides ABA treatment, particularly *ATHB7 *and -*12*, which showed 12 to 25 times upregulation [65]. However, *ATHB1*, to which the present *HDZip *finds maximum homology, have been found to be down regulated upon NaCl treatment [62]. The response of a member of a family of genes to the external environment could be, however, a species-specific phenomenon as *CPHB-6 *and *CPHB-7 *(*Craterostigma plantagineum *HDZip genes) upregulated upon ABA treatment [66], also finds maximum homology with *ATHB-1*. Hence, the HDZip genes overexpressed in the present case with a combined ESTs redundancy of 7.52% could be very important from the point of view of salt tolerance in plants.

The role of the other transcription factors in salt tolerance processes may not be ruled out as the genes of at least two more of them, C2H2 zinc finger (C2H2-ZnF) and white collar (WC1), were overexpressed (Table 2). Among them, the C2H2 type zinc finger protein with 176 members in *A. thaliana *[67] and 189 members in *O. sativa *[68], constitute one of the largest families of transcriptional regulators in plants. These are mostly plant specific, and synthesis of many of them has been found to be enhanced under salt [69-71] and other environmental stresses [70,71]. The importance of the protein in salt tolerance is also substantiated from the fact that tobacco plant transgene for C2H2-ZnF (*ZFP182*, overexpressing in rice under salt stress) showed increased tolerance to salt stress [69]. With regard to WC-1, however, no report is available so far indicating its possible involvement in salt or abiotic stress tolerance. The protein is only known to mediate blue-light and circadian response [72]. The gene has so far not been found to be overexpressing under salt or other environmental stresses. Besides, the overexpression of the gene in *Neurospora crassa *does not result in any upregulation of the genes reportedly involved in salt or abiotic stress tolerance [72]. There is also no report of any abiotic factor accelerating accumulation of pasticcino-1 (PSA1), which regulates the function of NAC-like transcription factors by controlling its targeting to nucleus [73]. However, the NAC family of transcription factors, which is one of the largest transcription factor families in plant genomes, have not only been implicated in plant development [73,74], but also in various abiotic stress responses [75]. Hence, it is plausible that PSA1 might be important from the point of view of salt tolerance depicting that a lots of cellular changes might be necessary for a plant to grow and perform under salt stress.

Overexpression of the genes encoding protein with various functional domains, such as CBS, F Box, C2 and C3H4 (Table 2) mediating important biochemical processes, mainly protein modification, degradation and membrane trafficking of proteins, is suggestive of their important role in adaptation of cells to NaCl enriched environment. In this regard, peroxin (PEX), a protein containing C3H4 Zn-RING finger, has been found to be involved in biogenesis of peroxisomes [76] important for not only carrying out fatty acid β-oxidation for energy generation, but also for protecting photo-damage of the photosynthetic machinery by carrying out photo-respiration. Besides, the organelle also harbour an antioxidant enzyme, catalase, required for eliminating H_2_O_2 _generated in plants under metabolic stress induced by NaCl [20], which otherwise would lead to oxidative damage to the cells. A RING finger containing protein (Rbx1) also forms a part of ubiquitin-proteosome system responsible for degrading the regulatory and misfolded proteins [77]; Rbx1 mediates binding of ubiquitin carrier protein (E2) to the multi-subunit ligase (E3) comprising of Skp1, culling1 and F-box protein (SCF), and the F-box subunit of E3 then recruits the protein to be poly-ubiquitinated and subsequently degraded [77,78]. Overexpression of the genes encoding proteins with C3H4 and F-box motif in the plant in response to NaCl stress thus indicated enhanced synthesis of regulatory proteins, which are possibly destined to be degraded after their role in the adaptive processes are over.

The precise function of CBS (Cystathione-β-synthase) domain protein is yet to be understood, although thought to be regulatory. Overexpression of the gene encoding CBS domain containing protein and its presence in AMP activated protein kinase (AMPK), the cellular energy sensor, nevertheless, does suggest that salt adaptation could be linked to energy metabolism. In fact, it has been reported that CBS domain in AMPK has greater affinity for AMP than for ATP, and as the cellular energy content drops (low ATP, high AMP), binding of AMP to CBS domain of AMPK facilitates its phosphorylation making the enzyme active [79]. Once activated, AMPK drives the metabolic pathway towards ATP accumulation [79]. Besides, CBS domain is also present in plants in various chloride channels, which open upon binding of ATP to the domain, and thus it could be important from the point of view of regulation of membrane potential, Cl^- ^homeostasis and osmotic adjustment in plants under NaCl stress [80].

The overexpression of the genes encoding various transcription factors under NaCl stress in *S. maritima *is no doubt suggestive of great metabolic changes that might be occurring in plants depending upon their need for survival and growth under salt stress. However, these changes are not possible until the stress signal is perceived. This is supported in part by the overexpression of the gene encoding protein with C2 domain, a Ca^2+ ^binding motif. Besides having affinity for Ca^2+^, C2 domain also displays remarkable property of recruiting a variety of other ligands and substrates, such as phospholipids and inositol phosphate [81]. Multiple copies of C2 domains have been identified in a growing number of eukaryotic signalling proteins that interact with cellular membranes and mediate a broad array of critical processes, including membrane trafficking, activation of GTPase for vesicular trafficking, control of protein phosphorylation and generation of lipid second messenger involved in signal transduction [81,82]. The Ca^2+^-dependent tolerance of plants to NaCl [20,83] could in fact be a result of enhanced synthesis of Ca^2+^-binding domain containing proteins. However, no known Ca-binding proteins, like Ca^2+^/calmodulin dependent protein kinase PsCCaMK, the *Arabidopsis *protein AtPC1, the membrane associated protein in rice OsEFA27 and *Arabidopsis *RD20, etc. was found to be overexpressed in the present case. The stress signal perception might also be G-protein mediated as overexpression of the gene encoding the protein (Transducin) was observed under NaCl stress in the present case; the involvement of G-protein in transduction of environmental signal is well documented [84]. However, none of the effectors in G-protein signalling was found to be overexpressed. The appearance of various phosphatases and kinases (Table 2, see Additional file 1), nevertheless, does suggest that many changes in the metabolic processes in response to external or internal signals must be mediated by protein phosphorylation and dephosphorylation.

Besides phosphorylation, O-linked β-N-acetylglucosamine (O-GlcNAc) modification of proteins could be abundant in *S. maritima *under NaCl stress, as it appears from the overexpression of O-GlcNAc transferase (OGT) gene (Table 2), and hence this could be an important biochemical event in the salt tolerance process. A large number of nuclear and cytosolic proteins are O-GlcNAc modified, and has been reported to affect stability of proteins and their sub-cellular localization [85]. One mechanism by which O-GlcNAc addition affect the changes in protein activity is through competition between O-GlcNAcylation and phosphorylation for the modification of serine/threonine residues. In fact, reciprocal phosphorylation/O-GlcNAcylation of specific amino acid has been demonstrated for several proteins, including the transcription factor, c-myc, and the reciprocal modification was found to differentially affect the activities of these proteins [86]. However, not all the substrate proteins are regulated via reciprocal phosphorylation/O-GlcNAcylation. In some cases, O-GlcNAc addition may directly affect the protein activity [87]. Although there is no report of *OGT *overexpression under any environmental stress, OGT activity has been found to be essential for plant survival [87].

The salt adaptive metabolic changes could be mediated by the heat shock protein HSP70, a well known molecular chaperon. This is reflected from the overexpression of the genes encoding Bcl2 binding BAG and DnaJ proteins (Table 2), which physically interact with HSP70 [88,89]. DnaJ like proteins are involved in a variety of processes including protein folding, protein partitioning into organelles, signal transduction and targeted protein degradation. Moreover, the DnaJ domain of the protein has especially been shown to interact directly with HSP70, thereby regulating its ATPase activity, which affects protein binding and folding [89]. Similar to DnaJ protein, BAG protein also has a conserved domain (BAG domain) to interact with the heat shock protein (HSP70/HSC70). Hence, the BAG protein might also be involved in protein folding and maturation [90]. The increased synthesis of BAG protein protects various cell types from heat-induced apoptosis, possibly through interaction with HSP70 and HSP40 [91].

In addition to the post translational events, pre-translational processes like mRNA and tRNA processing, and the translational event itself appear to be greatly changed or adjusted to suit the requirement demanded by salt adaptive physiological processes. This is evident from the significant increase in pre-mRNA splicing factor, 60S ribosomal P0 protein, appr-1p processing enzyme family protein, eukaryotic elongation factor 1A, translation initiation factor 2B-β sub-unit and valyl-tRNA. However, little is known about the role of these proteins in salt adaptation, or abiotic stress adaptation in general.

At the physiological level, the NaCl adaptive response was highly visible in terms of overexpression of the genes of many enzymes related to the synthesis and accumulation of glycinebetaine (Table 3). The most important among them being *PEAMT *mediating the conversion of phosphoethanolamine to phosphocholine, which is either dephosphorylated to form choline directly [92] or first incorporated into phosphatidylcholine and then metabolized to choline [93] (Fig. 2). Primarily the synthesis of choline occurs following the route phospho-ethanolamine (P-EA) to phospho-choline, P-choline (bold arrows, Fig. 2). However, the route P-EA to phosphatidylcholine, Ptd-choline (normal arrow, Fig. 2) also contributes substantially to choline synthesis depending upon the species [94]. The synthesis of the compound by other routes (broken arrows, Fig. 2) is also possible [94]. The choline produced in the cytoplasm is transported to the chloroplast where it is converted to glycinebetaine by the reactions catalyzed sequentially by choline monooxygenase (CMO) and betainealdehyde dehydrogenase (BADH). Thus, PEAMT is although not directly involved in the synthesis of glycinebetaine, the enzyme appears to be very important in the biochemical pathways of synthesis of the osmoticum. The activity of PEAMT has been reported earlier to be greatly enhanced in the betaine accumulating halophyte *Atriplex nummularia *[95] and glycophyte spinach [92] by salt stress. These, together with the overexpression of *PEAMT *in the present case with high ESTs redundancy (Table 3) suggest that increased synthesis of choline could be highly essential for the survival of plants under salt stress, particularly those accumulating glycinebetaine, and that *S. maritima *might be a glycinebetaine accumulating halophyte. However, no overexpression of the gene encoding BADH, the enzyme catalysing conversion of betainealdehyde to glycinebetaine (Fig. 2), the final step of glycinebetaine synthesis, was seen in the plant in response to the NaCl treatment (Fig. 4), although this has been reported for terrestrial glycophyte as well as halophyte [96,97]. This could be because the availability of choline is probably more important for the accumulation of glycinebetaine than the amount of the enzymes catalysing the conversion of choline to glycinebetaine, i.e. choline monoxygenase (CMO) and BADH (Fig. 2). The fact is substantiated from the observation that the supply of exogenous choline leads to glycinebetaine synthesis even in the plants not accumulating glycinebetaine naturally, like *Arabidopsis thaliana*, *Brassica napus *and *Nicotiana tobacum *[98]. Moreover, modelling of the labelling kinetics of choline metabolites upon supply of ^14^C-choline demonstrated that choline import into chloroplast indeed limited its flux to glycinebetaine [99]. Hence, it was postulated that a high-activity choline transporter in the chloroplast envelope could be an integral part of glycinebetaine synthesis pathway in the species that accumulate the compound naturally [99]. The overexpression of three isoforms of choline transporter gene, each with high EST redundancy, in the present study appears to support the hypothesis.

The fact that choline synthesis is really enhanced upon salt treatment is supported from the enhancement in the expression of the gene encoding SAM synthesizing enzyme, S-adenosylmethionine synthase, SAMS (Table 3, Fig. 2) , which uses methionine and ATP as substrates. SAM is consumed in the glycinebetaine synthesis pathway for SAM-dependent methylation of ethanolamine (EA) or phosphoethanolamine (P-EA) in successive steps to produce choline, phosphocholine or phosphatidylcholine (Fig. 2). Besides, SAM is an essential substance for the living cells as a methyl group donor and as a precursor in ethylene biosynthesis catalyzed by ACC synthase and ACC oxidase (Fig. 2) [53,100]. Hence, maintaining a considerable pool of SAM by enhancing the rate of its synthesis must be essential when the physiological condition so demand, as in the case of glycinebetaine accumulation under salt stress. In fact, it has been observed that in halophyte *Atriplex nummularia *accumulating glycinebetaine under salt stress, the transcript levels of *SAMS *co-regulates with that of *PEAMT *in response to varying salinity level [95]. The present work thus indirectly suggests that while going for the development of transgenic plant for enhanced accumulation of glycinebetaine, the attention should be focused on increasing the level of choline and its transport to chloroplast. Attention should also be paid to the fact that the transfer of the methyl group from SAM generates S-adenosyl-L-homocysteine (SAH), which is a potent inhibitor of SAM dependent methyltransferases. Hence, SAH should be hydrolysed or removed, and this is done by S-adenosylhomocysteine hydrolase (SAHH), breaking it into homocysteine and adenosine [101]. The overexpression of *SAHH *in *S. maritima *under salt stress in the present case is an indication that the plants accumulating glycinebetaine should overcome SAH accumulation, and that the plants transgenic for enhanced production of choline should also show enhanced expression of *SAHH*.

Besides glycinebetaine, proline is another osmoticum widely reported to accumulate in plants under salt stress. However, the report of accumulation of both glycinebetaine and proline in a plant in response to salt stress is limited [102]. The overexpression of the gene encoding P5CS (Table 3), the enzyme catalysing the conversion of Δ^1^-pyrroline-5-carboxylate to proline, the final step in the conversion of glutamate to proline, nevertheless, does suggest that proline, in addition to glycinebetaine, might be accumulating in the plant under NaCl-stress. This may in fact be the requirement as the accumulation of glycinebetaine remains restricted to the chloroplast (Fig. 2), and hence the osmotic adjustment of the cytosol might be achieved by the accumulation of proline. Significant increase in the activity of P5CS (Fig. 5a), besides the expression of its gene (Fig. 3, 4), also indicated possible accumulation of proline in the plant in addition to glycinebetaine upon salt treatment.

Although the maintenance of cellular ionic homeostasis has been emphasized for the survival of organism, especially under ionic stress [7], no overexpression of the genes of any known cation transporters, particularly of the alkali cations, was observed in the present study, except of a putative Na^+^/H^+ ^antiporter of low E value (Table 3). The finding is in contrast to the report of overexpression of Na^+^/H^+ ^antiporter gene in several plant species under salt stress [7,8]. Moreover, no Ca-binding protein or protein kinase was identified in the present study, in contrast to the SSH study in tomato [18], suggesting the absence of the SOS (salt overly sensitive) signalling pathway of Na^+ ^efflux in the halophytes like *S. maritima*. Highly enhanced expression of the genes encoding at least two proteins (FC932784 and FG228211) finding high homology with the proteins conceptually translated as cation-efflux transporters from *A. thaliana *genome database, nevertheless, does suggest important role of cation efflux in salt tolerance, although the ion(s) they transport remains to be identified.

Several genes having no known relationship with salt tolerance were found to be overexpressing in the plant in response to the salt treatment. The two well known among them are that encoding CCL (CCR-like, cold circadian rhythm-RNA binding like) protein and carbonic anhydrase (CA). CCL gene encodes highly unstable mRNA, the stability being regulated by circadian clock [103]. The transcript of this gene is significantly more stable in the morning than in the afternoon [103]. However, the EST redundancy of the CCL gene (Table 3) in the present study indicated high accumulation of transcripts of the gene even in the evening (the plants for the isolation of RNA were harvested in the evening). Hence, it appears that the salt treatment either had increased the stability of the *CCL *transcripts or had enhanced the expression of the gene in the plant. Although the role of the RNA binding proteins in posttranscriptional regulation of gene function, critical for eukaryotic growth and development, is well documented [104], expression of none of the genes encoding these proteins, including *CCL*, has been reported to be affected by salt treatment. The physiological function of carbonic anhydrase on the other hand is well known, facilitating CO_2 _availability for photosynthesis in C_4 _and submerged aquatic plants [105-107]. The expression of its gene has also been reported to be highly enhanced in plants in response to salt treatment [29]. Besides, *Arabidopsis *plant transgenic for rice carbonic anhydrase (*OsCA1*) has been demonstrated to show greater salt tolerance than the wild type at the seedling stage [29]. However, any physiological or biochemical role of the enzyme in salt tolerance is yet to be established, especially in the non-aquatic angiosperm where the availability of CO_2 _is not influenced by salinity. The tolerance of *Dunaliella salina*, a unicellular alga, to nearly saturating NaCl concentration, nonetheless, has been suggested to be in part due to increased accumulation of a halophilic plasma membrane CA isoform. The enzyme shows maximum activity at much higher NaCl concentration and is much more resistant to inhibition by salt than the enzyme isolated from the salt-sensitive alga *Chlamydomonas reinhardtii*; the unique characteristics of *D. salina *carbonic anhydrase potentially enable the enzyme to optimise inorganic carbon utilization in high salinities [105].

Xyloglucan endotransglycosylase/hydrolase (*XTH*) and expansin-3, both involved in cell wall metabolism, are also among the genes that have no biochemically or physiologically known relationship with salt tolerance, but were overexpressed upon salt treatment of the plant in this study (Table 3). One of them, *XTH*, a glucan endo-1,3-β-glucosydase, has also been reported to be greatly overexpressed in tomato upon salt treatment [18]. XTH catalyses endo cleavage of xyloglucan polymers and subsequent transfer of the newly generated reducing ends to other polymeric or oligomeric xyloglucan molecules and thereby participates in cell wall formation and elongation [108]. Expansin-3 on the other hand belongs to a group of extracellular non-enzymatic cell wall protein, which loosens the linkage between cellulose microfibrils by modifying the cell wall matrix in terms of increasing the mobility of the constituent matrix polymers [109]. The modification allows the cell wall to yield to the tensile stress created in the wall by the turgor pressure. Enhanced expression of XTH and expansin-3 in the present study seems to be in agreement with the visibly healthy growth and flaccid leaves of the plant grown in the saline medium than that grown without salt. However, no gene encoding expansin has so far been reported to be overexpressing in response to salinity. The maintenance of a greater leaf turgidity in the plant grown on salt than that grown without salt could be by accumulation of osmolytes, as discussed above.

An important physiological event that is not found in animals is photorespiration, which occurs in many plants upon their illumination leading to breakdown of rubisco-1–5-biphosphate and synthesis of glycolic acid in the chloroplast. The glycolic acid produced is oxidized to glyoxalic acid in the peroxisomes with concomitant generation of H_2_O_2_. Overexpression of the gene encoding glycolate oxidase (see Additional file 1) does suggest enhancement in photorespiration, and catalase (Cat) is probably synthesized at enhanced rate (Fig. 5b, c, see Additional file 1) to protect the plant from oxidative damage by the accumulating H_2_O_2_. However, so far no relationship between photorespiration, or any of its components, and salt tolerance has been reported.

From the functional characterization of the unigenes, and redundancy of EST in the individual group, it appears that the proteins involved in cell cycle and DNA processing must be playing crucial role in salt adaptation as the redundancy of ESTs in the group was very high, 13.6% (Fig. 7, sub-category C), despite very low contribution by the unigenes (Fig. 6). In the same way, the proteins involved in transcription (sub-category D) and protein synthesis (sub-category E) must also be very important in supporting the plant to go through the salt adaptation processes. This is because the transcription of the genes in these categories greatly increased upon NaCl treatment and contributed individually 7–9% of the ESTs population (Fig. 7) in contrast to each sub-category representing 1.8% of the total unigenes (Fig. 6). The role of the proteins regulating protein activity (sub-category H), however, seems to be very important as the size of the ESTs of this category (Fig. 7) was more than 10 fold the size of the unigenes (Fig. 6) in the group. Besides, the ESTs of the proteins involved in signal transduction (sub-category J) also increased significantly compared to the size of the unigenes in the group. The results thus strongly suggest salt tolerance to be heavily dependent on the expression of the genes contributing to the information pathway (sub-categories C-H) of the plant protein functional catalogue involving protein controlling important cellular functions, such as cell cycle, transcription, protein synthesis, regulation of protein activity, etc. (Fig. 6, 7). Besides, the proteins involved in the developmental processes like cell type differentiation (sub-category Q), biogenesis of cellular components (sub-category P), etc. also appear to play important role in salt tolerance as the EST abundance of the related genes was found to be considerably high after those of the genes of the information pathway. Nonetheless, importance of the other proteins, particularly the unknown ones (sub-category U), cannot be ignored as many biochemical processes determining salt tolerance might be hidden in this pool, although the EST redundancy of the genes encoding these proteins was found to be much less than the size of unigenes in the group.

## Conclusion

The present study thus reports for the first time differential expression of genes under salt stress in a natural halophyte. From the number of the unigenes showing overexpression in the plant in response to the salt application, it is highly convincing that the salt tolerance process is highly complex. However, even more puzzling is that a species differs greatly from the other in the genes that are salt regulated, as revealed by a comparative analysis of the present data with that available for rice [19] and tomato [18]. Therefore, it is desirable that more data are generated on the salt inducible/responsive genes of various plant species, particularly of the halophytes in order to identify the key elements involved in the salt tolerance processes, which is not possible with the currently available SSH cDNA libraries related to salt responsive genes. Nevertheless, it is quite convincing that the genes/proteins involved in the flow of information and developmental processes could be of much importance in salt stress response and adaptation of plants to saline environment. The number of genes possibly involved in salt tolerance can be narrowed down further if the SSH cDNA library data on salt inducible genes are available for a sufficiently large number of closely related halophytic species. Once this is achieved, the full length cDNA of the desired genes can be obtained easily and their specific functions in salt tolerance can be investigated further in suitable model systems using the available genetic transformation and gene knockdown/kockout technologies. Functional characterization of the proteins/genes that overexpress under salt stress, but have no known function, will be of particular importance in understanding the complex mechanism associated with salt tolerance. This will eventually enable the biotechnologists to target the right gene(s) for the genetic engineering of crop plants for improved salt tolerance. Nevertheless, the success of such effort will largely depend on the critical number of genes that must be genetically engineered to make a crop plant salt-resistant, besides on the effect of the transformation on the yield quantity and quality of the crop.

## Authors' contributions

BPS conceived and coordinated the study. BBS carried out most of the molecular biology work. BPS did the enzyme activity studies. BPS and BBS jointly analysed the data and drafted the manuscript. BPS critically revised the manuscript for intellectual content. Both the authors read and approved the final manuscript.

## Supplementary Material

Additional file 1 **BLASTX results of the ESTs sequences of NaCl-induced genes in *S. maritima***. mRNA was isolated from the leaves of young *S. maritima *plants exposed to 425 mM NaCl for 24 h (treated) and that not treated with NaCl (control). Forward SSH cDNA library, representing salt-induced genes, was constructed considering mRNA from the NaCl-treated plant as 'Tester' and that from control as 'Driver'. cDNAs of the library were cloned and transformed, and 502 ESTs from such clones were sequenced. ESTs sequences from the forward SSH cDNA library of *S. maritima *were grouped into singletons and contigs using TIGR Assembler  and were termed as unigenes. The unigene sequences were blasted for homology search using BLASTX programme (default) at NCBI database. The search results are given. EST redundancy of each unigene is also given along with the average size of the ESTs constituting the unigene. Click here for file
